# MMP-9 Cleaves SP-D and Abrogates Its Innate Immune Functions *In Vitro*

**DOI:** 10.1371/journal.pone.0041881

**Published:** 2012-07-30

**Authors:** Preston E. Bratcher, Nathaniel M. Weathington, Heidi J. Nick, Patricia L. Jackson, Robert J. Snelgrove, Amit Gaggar

**Affiliations:** 1 Department of Medicine, University of Alabama at Birmingham, Birmingham, Alabama, United States of America; 2 Division of Pulmonary, Allergy, and Critical Care Medicine, Department of Medicine, University of Pittsburgh Medical Center, Pittsburgh, Pennsylvania, United States of America; 3 National Heart and Lung Institute, Imperial College London, London, United Kingdom; 4 Gregory Fleming James Cystic Fibrosis Research Center, University of Alabama at Birmingham, Birmingham, Alabama, United States of America; 5 University of Alabama at Birmingham Lung Health Center, University of Alabama at Birmingham, Birmingham, Alabama, United States of America; 6 Medicine Service, United States Department of Veterans Affairs Medical Center, Birmingham, Alabama, United States of America; University of Bern, Switzerland

## Abstract

Possession of a properly functioning innate immune system in the lung is vital to prevent infections due to the ongoing exposure of the lung to pathogens. While mechanisms of pulmonary innate immunity have been well studied, our knowledge of how these systems are altered in disease states, leading to increased susceptibility to infections, is limited. One innate immune protein in the lung, the pulmonary collectin SP-D, has been shown to be important in innate immune defense, as well as clearance of allergens and apoptotic cells. MMP-9 is a protease with a wide variety of substrates, and has been found to be dysregulated in a myriad of lung diseases ranging from asthma to cystic fibrosis; in many of these conditions, there are decreased levels of SP-D. Our results indicate that MMP-9 is able to cleave SP-D *in vitro* and this cleavage leads to loss of its innate immune functions, including its abilities to aggregate bacteria and increase phagocytosis by mouse alveolar macrophages. However, MMP-9-cleaved SP-D was still detected in a solid-phase *E. coli* LPS-binding assay, while NE-cleaved SP-D was not. In addition, MMP-9 seems to cleave SP-D much more efficiently than NE at physiological levels of calcium. Previous studies have shown that in several diseases, including cystic fibrosis and asthma, patients have increased expression of MMP-9 in the lungs as well as decreased levels of intact SP-D. As patients suffering from many of the diseases in which MMP-9 is over-expressed can be more susceptible to pulmonary infections, it is possible that MMP-9 cleavage of SP-D may contribute to this phenotype.

## Introduction

Of the more than twenty matrix metalloproteases, matrix metalloprotease 9 (MMP-9) is one of the most well studied, both *in vitro* and *in vivo*, within the context of normal and several disease states. Also known as 92-kDa gelatinase, or gelatinase B, MMP-9 has a variety of substrates and roles in functions as diverse as tissue remodeling, tissue repair, and regulation of inflammation [1]. More than 30 substrates have been discovered for MMP-9, including gelatin, type IV and V collagens, TNF-α, a variety of surface receptors [including vascular endothelial growth factor receptor-2, beta2-adrenergic receptor, and ICAM-1 (reviewed in [2]], protease nexin-1, interleukin (IL) 8, IL-1β, and leukemia inhibitory factor [3], [4], [5], [6], [7]. MMP-9 has a structure similar to other matrix metalloproteases, and includes four domains: an N-terminal signal sequence, a pro-domain region, an active catalytic domain, and a hemopexin-like C-terminal domain (reviewed in [8]). The prodomain region must be cleaved in order for the protein to become active.

Due to its many functions in the body, MMP-9 dysregulation has been implicated in a variety of diseases. MMP-9 expression and activation levels have been shown to be elevated in lower airway secretions and/or serum of smokers and patients with chronic obstructive pulmonary disorder (COPD), emphysema, idiopathic pulmonary fibrosis (IPF), acute respiratory distress syndrome (ARDS), cystic fibrosis (CF), and asthma after allergen challenge [9], [10], [11], [12], [13], [14], [15], [16], [17], [18], [19]. Whilst the role that MMP-9 plays in these conditions is not fully understood, emerging evidence from *in vitro* studies and animal models suggests that MMP-9 may be contributing to the pathogenesis of these disorders rather than being a response to them (reviewed in [1]). An increase in cleavage of various MMP-9 substrates can result in an increase in inflammation *in vitro*, and inflammation *in vivo* results in alterations in clinical outcomes in patients with many of these pulmonary conditions.

MMP-9 expression is also altered during infections by several different pathogens. MMP-9 is increased in bronchoalveolar lavage fluid during hospital-associated pneumonia and *Pseudomonas aeruginosa* infections, in serum during *Mycobacterium tuberculosis* and *Helicobacter pylori* infections, and in cerebrospinal fluid during *Klebsiella pneumonia* meningitis [20], [21], [22], [23]. Although MMP-9-deficient mice are more susceptible to systemic Streptococcal infections and *Escherchia coli* peritonitis, the knockouts are less susceptible to pulmonary *Francisella tularensis* and urogenital *Chlamydia muridarum* infections [24], [25], [26], [27]. It is, therefore, likely that MMP-9 has a variety of functions in host immune defense. Interestingly, patients with many of the lung diseases in which MMP-9 levels are elevated are also more susceptible to pulmonary infections, such as in COPD and CF.

One molecule in the lung that also has roles in regulation of inflammation and the innate immune defense is Surfactant Protein D (SP-D) (reviewed in [28], [29]). In addition to these roles, SP-D is also involved in surfactant homeostasis. It belongs to the collectin family of pathogen recognition receptors, and its structure consists of two main regions: a collagen-like domain and a carbohydrate recognition (or lectin) domain (CRD). It is able to bind to a variety of pathogens through this CRD. *In vivo*, SP-D is found in homotrimers which are associated through their collagen-like domains, and four homotrimers can further associate to form a cruciform-like dodecamer. It is through this multimerization that SP-D is able to cause the formation of aggregates of pathogens and allergens (reviewed in [30]).

Similar to MMP-9, SP-D is thought to be involved in the mechanism behind the pathology of many pulmonary diseases, including asthma, smoking-associated respiratory problems, COPD, IPF, and cystic fibrosis. The concentration of SP-D is increased in the serum of COPD, IPF, and CF patients, increased in the lungs of asthmatics after allergen challenge, and decreased in the lungs of smokers [31], [32], [33], [34], [35], [36]. Its roles in these diseases are likely due to its functions in surfactant homeostasis as well as regulation of inflammation. SP-D has also been found to be important in the pulmonary defense against many pathogens, and is able to directly interact with viruses, fungi, and bacteria (reviewed in [30]). SP-D knockout mice have been shown to be more susceptible to respiratory infections with *Streptococcus pneumoniae*, *Pseudomonas aeruginosa*, *Pneumocystis carinii*, *Influenza A* virus, Respiratory syncytial virus, and *Aspergillus fumigatus* [37], [38], [39], [40], [41], [42].

Due to the overlap of alterations in MMP-9 and SP-D in pulmonary diseases and infections, we sought to examine the effects that MMP-9 has upon SP-D *in vitro*. We hypothesized that SP-D may serve as a substrate for MMP-9 as SP-D contains a collagen-like region and MMP-9 has the ability to cleave collagen. Experiments were performed to examine interactions between purified recombinant proteins, and several *in vitro* assays demonstrate the ability of MMP-9 to abrogate the innate immune functions of SP-D.

## Results

### MMP-9 Cleaves SP-D

**Figure 1:**
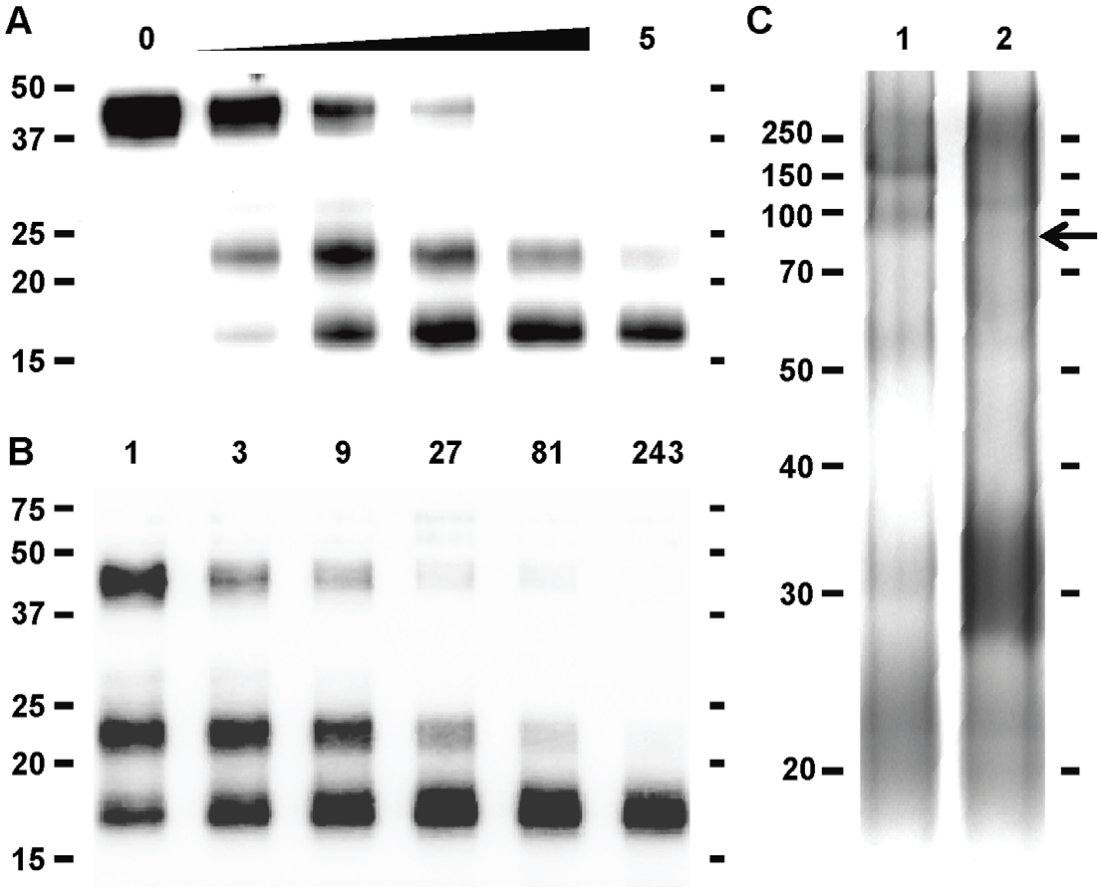
MMP-9 Cleaves SP-D in a Dose- and Time-dependent Manner. (A) Dose and (B) time course digestions of SP-D by MMP-9. For the dose course (A), the reaction loaded onto lane 2 contained 62 ng/mL MMP-9 and the concentration was raised 3-fold serially to 5 µg/mL MMP-9 at lane 6. Reactions were incubated for 4 hours. For the time course (B), the length of digestion in minutes is listed above the lane. SP-D concentration was 20 µg/mL for both experiments, and MMP-9 concentration was 5 µg/mL for the time course. For the Native PAGE (C), lane 1 contains intact SP-D and lane 2 contains cleaved SP-D. An arrow indicates the band corresponding to MMP-9.

To determine if SP-D could be cleaved by MMP-9, recombinant MMP-9 and recombinant SP-D were incubated together at 37°C. Intact recombinant SP-D monomers were detected as a ∼43 kDa band using SDS-PAGE under reducing conditions (with minor ∼27 and ∼23 kDa bands). Incubation with MMP-9 led to the formation of two prominent bands at ∼23 and ∼17 kDa (Figure 1). The relative amount of the ∼23 kDa band and the presence and relative amount of the ∼17 kDa band were both dependent on dose of MMP-9 and the time of incubation. Based on the dose curve (Figure 1A), 20 µg/mL of SP-D was incubated with 5 µg/mL of MMP-9, which resulted in the formation of the 23 and 17 kDa fragments after only one minute. After four hours, all of the SP-D detected had been degraded from its intact, 43 kDa form into the smaller fragments. In addition, after extensive cleavage, the 23 kDa band decreased in intensity relative to the 17 kDa band, suggesting that the 23 kDa fragment may be further digested by MMP-9 (Figure 1B). In order to further analyze the effect of cleavage on the structure of SP-D, we examined intact and cleaved SP-D using Native PAGE (Figure 1C). The results reveal that there is a significant alteration of the multimeric structure of SP-D after incubation with MMP-9, as changes in the banding patterns were observed.

### SP-D-mediated Bacterial Aggregation is Disrupted by MMP-9 Cleavage

**Figure 2:**
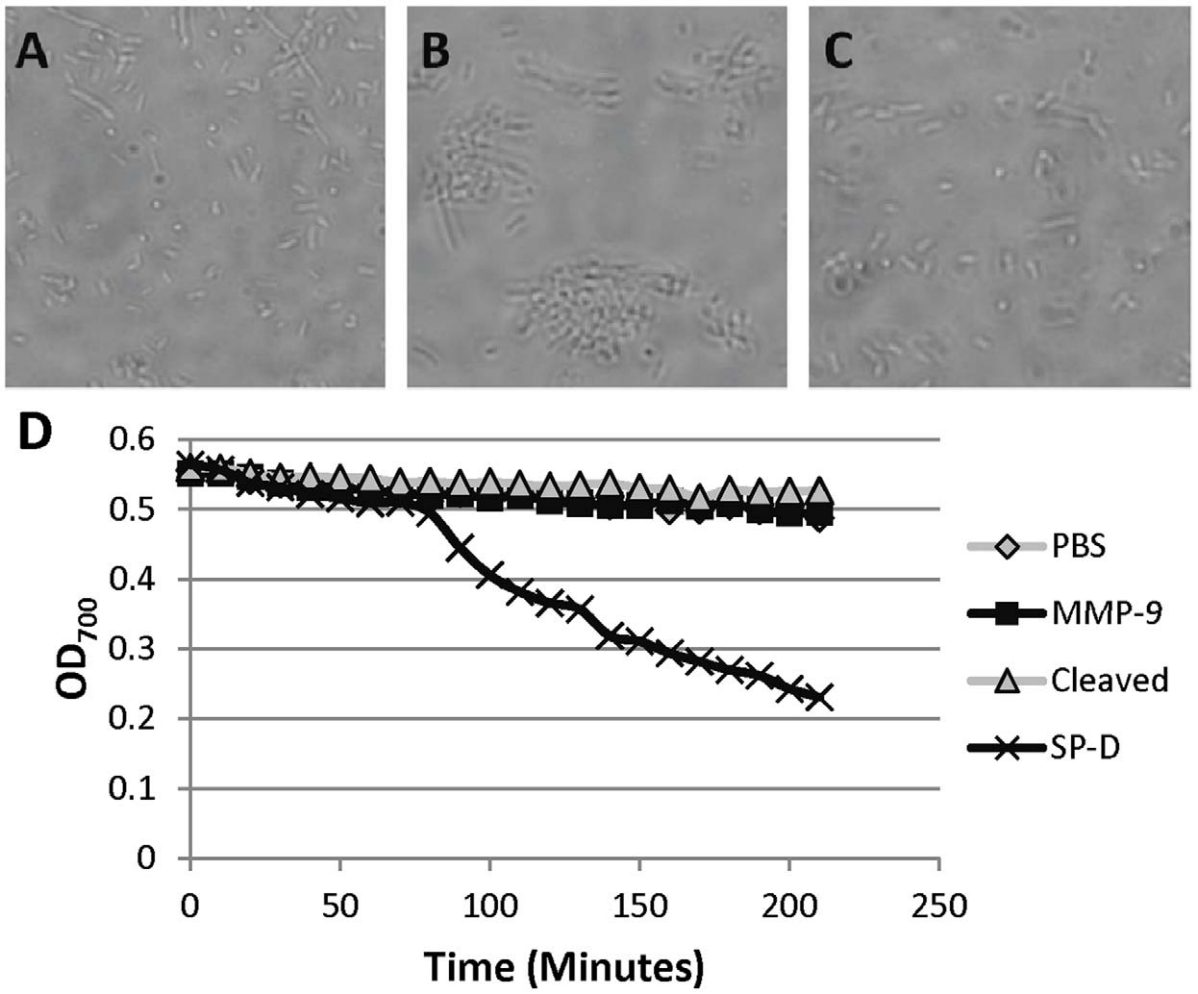
Cleaved SP-D Fails to Agglutinate *E. coli*. An equal volume of concentrated bacteria and (A) PBS, (B) 1 µg/mL intact SP-D, or (C) 1 µg/mL cleaved SP-D were mixed and examined using brightfield microscopy. Original magnification was 1000X. (D) Agglutination was quantitated using by monitoring the OD_700_ over time as previously described [50].

Incubation with SP-D *in vitro* results in the agglutination of a variety of pathogens, including *E. coli* [30]. In order to examine the effects of cleavage by MMP-9 on this function, we incubated *E. coli* with intact and cleaved SP-D (Figure 2). While incubation with intact SP-D led to the formation of aggregates of *E. coli*, incubation with cleaved SP-D or PBS had no visual effect upon the *E. coli* (Figure 2A–C). Incubation with MMP-9 alone also had no visual effect (not shown). In order to confirm these results in a more quantitative matter, we monitored the optical density (OD) of suspensions of *E. coli* with or without intact and cleaved SP-D, and only observed a decline in OD with intact SP-D (Figure 2D). Because multimerization of SP-D is necessary for the formation of bacterial aggregates after the CRD-mediated interaction of SP-D with the bacteria, we hypothesize that cleaved SP-D no longer agglutinates bacteria due to the fact that the multimers are disrupted.

### Loss of the Ability of SP-D to Increase Phagocytosis of *E. coli* by Alveolar Macrophages after MMP-9 Cleavage

**Figure 3:**
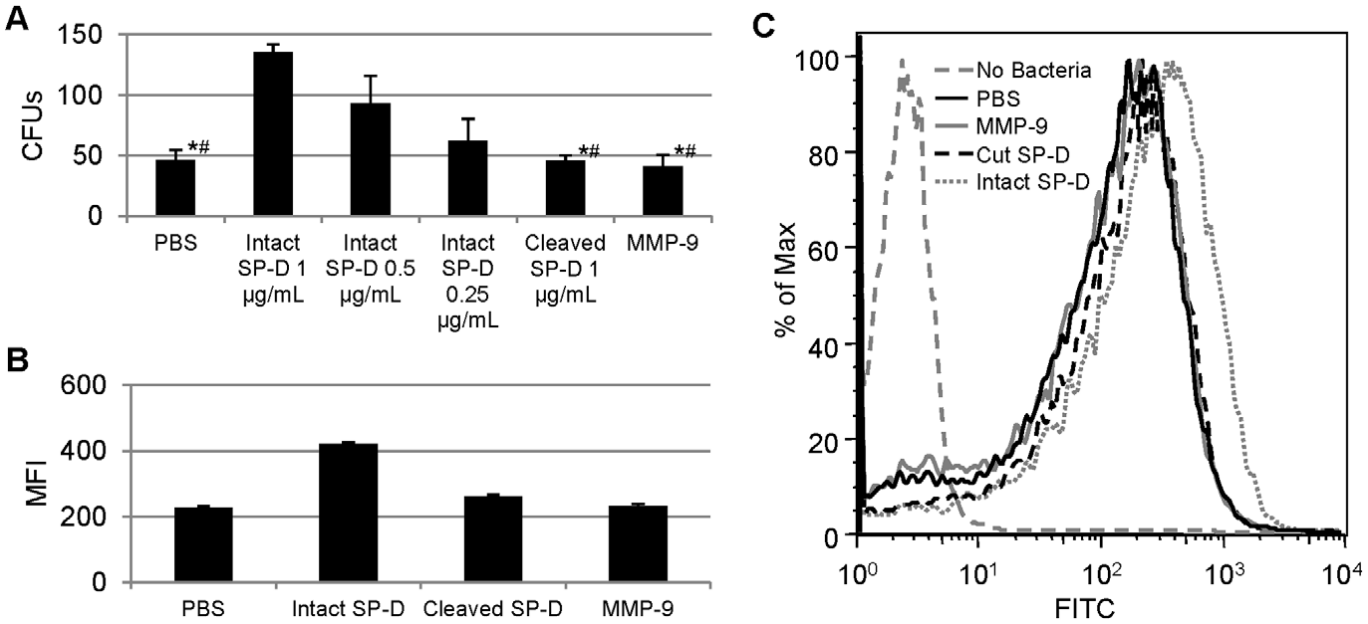
Cleaved SP-D Fails to Increase Phagocytosis by MH-S Cells. Examination and quantitation of phagocytosis of *E. coli* by MH-S cells was performed using (A) a gentamicin protection assay and (B and C) flow cytometry. For (A), all conditions are significantly different (p<0.05) from 1 µg/mL intact SP-D. An asterisk (*) denotes p≤0.001 when compared to 1 µg/mL intact SP-D. Columns marked with # are significantly different when compared to 0.5 µg/mL intact SP-D (p<0.05). For (B), intact SP-D causes MH-S cells to have significantly higher mean fluorescence intensity (MFI) than all other conditions (p≤0.001). Cleaved SP-D is significantly different when compared to both MMP-9 and PBS controls (p≤0.001). For (C), flow data was gated on forward and side scatter to select for MH-S cells. For both (A) and (B), error bars represent standard deviation.

In addition to agglutination, we also examined the ability of SP-D to serve as an opsonin for clearance of bound pathogens by host cells. For our *in vitro* model, we used a mouse alveolar macrophage cell line (MH-S). By adding *E. coli* preincubated with SP-D to these macrophages, we saw a dose-dependent increase in bacterial phagocytosis using a gentamicin protection assay (Figure 3A). After cleavage with MMP-9, the opsonic activity of SP-D was completely abrogated, and phagocytosis was decreased to levels comparable with the MMP-9 alone control. In addition to the gentamicin protection assay, we also used microscopy and flow cytometry to examine SP-D’s impact on phagocytosis of FITC-labeled *E.coli* (Figure 3B, C, and D). Results from these analyses confirmed those obtained using the gentamicin protection assay, demonstrating a striking loss of SP-D’s opsonic capacity following MMP-9 cleavage. Interestingly, the microscopy and flow cytometry results suggest that while the numbers of macrophages containing bacteria are very similar, those that do phagocytose bacteria contain more in the presence of intact SP-D.

### MMP-9–cleaved SP-D Retains CRD-dependent Binding to LPS

**Figure 4:**
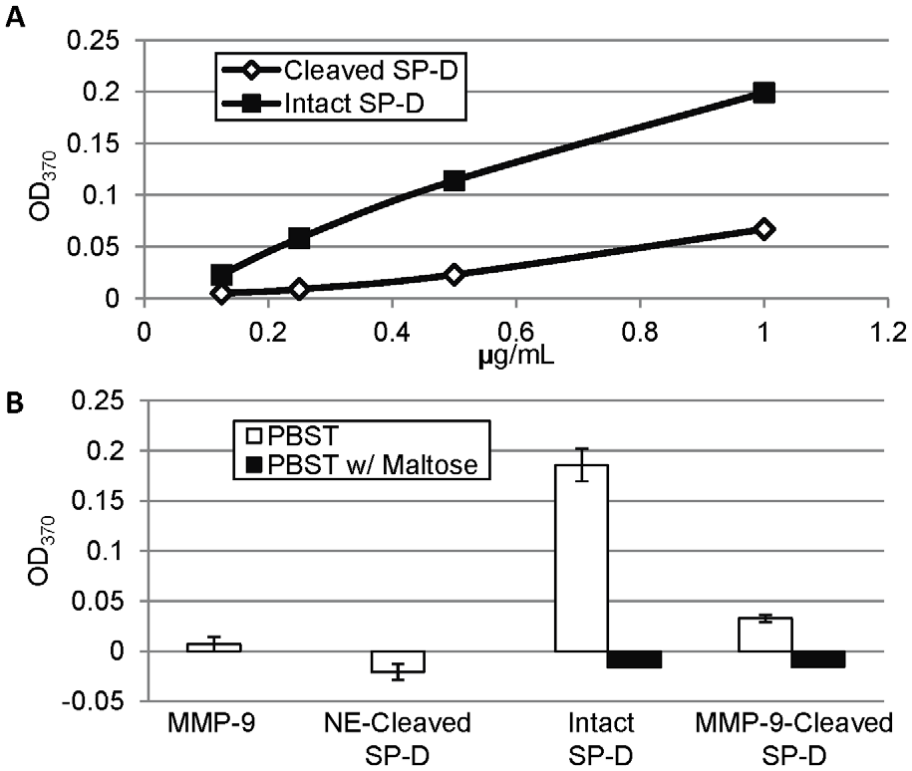
Cleaved SP-D Retains its Ability to Bind *E. coli* LPS. Examination of the ability of SP-D to bind to LPS-coated plates. In (A), the ELISA was performed using 2-fold serially diluted SP-D samples. In (B), 1 µg/mL intact or cleaved SP-D was analyzed in triplicate with MMP-9 as a control and NE-cleaved SP-D as a negative control. The average of PBST alone ran in triplicate was subtracted from all values. While intact and MMP-9-cleaved SP-D in PBST are significantly different from PBST alone (p≤0.001 and p<0.05, respectively) and NE-cleaved SP-D (p≤0.001 and p<0.05, respectively), MMP-9 along with intact and cleaved SP-D in PBST with maltose were not significantly different from PBST or PBST with maltose. For (B), error bars represent standard deviation.

In order to determine if the CRD of the fragmented SP-D retains its ability to bind to target molecules, we performed an ELISA using LPS-coated plates. Detection of both intact and cleaved SP-D was dose-dependent, suggesting that the cleaved SP-D was able to bind to LPS (Figure 4A). Although intact and cleaved SP-D were assayed at 1 µg/mL (Fig. 4B), there was a fivefold lower amount of MMP-9-cleaved SP-D detected; binding was completely abrogated by the addition of maltose, suggesting that this binding occurred through the CRD and that the CRD remained intact after cleavage by MMP-9. The decrease in the amount of SP-D detected when MMP-9-cleaved SP-D was assayed may be due to the fact that intact SP-D molecules are larger, and may bind more of the polyclonal antibody than the cleavage fragment containing the CRD; however, it possible that the cleavage may alter the ability of SP-D to bind LPS or the stability of this interaction. In contrast to MMP-9-cleaved SP-D, binding of NE-cleaved SP-D was not detected in this ELISA; this lack of CRD binding to carbohydrates by NE-cleaved SP-D has been previously shown by others [43]. This highlights differences in properties of the cleavage fragments generated by MMP-9 and NE. We additionally assayed for binding to plates coated with 5% BSA to control for SP-D binding to the blocking substance; although both intact and MMP-9-cleaved SP-D were detected, they were detected at levels significantly lower than that with LPS-coated plates (p<0.02 for intact and p<0.05 for MMP-9-cleaved, data not shown). These results indicate that both intact and MMP-9-cleaved SP-D seem able to bind to *E. coli* LPS, and this binding was inhibited by maltose, suggesting it occurs through the CRD.

### MMP-9 Cleaves SP-D more Efficiently than Neutrophil Elastase at Physiological Calcium Concentrations

**Figure 5:**
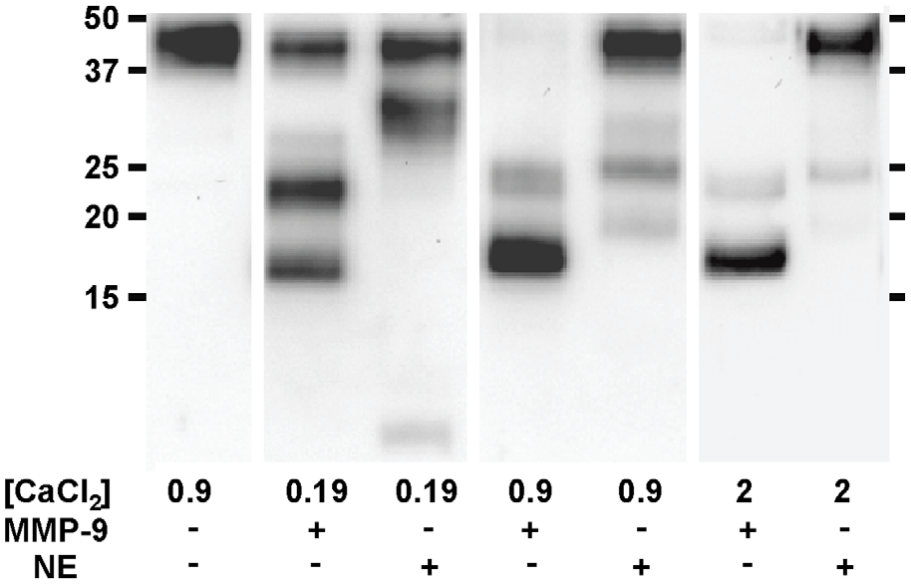
MMP-9 cleaves SP-D more potently than NE at higher calcium concentrations. Comparison of the fragments produced by cleavage with SP-D and NE. CaCl_2_ concentrations are listed in mM. For experiments at 0.19 mM CaCl_2_, incubations were carried out for 10 min at protease concentrations of 16.95 nM. For experiments at 0.9 and 2 mM CaCl_2_, incubations were carried out for 4 hours with or without protease at 60.24 nM.

Cleavage of SP-D by several serine proteases and Pseudomonal elastase has previously been well-described [43], [44], [45]. A study by Cooley et al. showed that of the serine proteases that cleave SP-D (which consist of neutrophil elastase (NE), cathepsin G, and proteinase-3), NE is the most potent [46]. However, several studies have reported that both the cleavage pattern and efficiency of cleavage by these serine proteases is dependent on the concentration of calcium [43], [46], [47]. As we did not expect calcium concentration to affect cleavage by MMP-9, we compared MMP-9 cleavage to NE cleavage of SP-D at a variety of calcium concentrations. As shown in Figure 5, while at low calcium levels of 0.19 mM, MMP-9 and NE cleave at comparable rates. But at 0.9 mM (the concentration used for all other experiments in this paper), MMP-9 is much more potent at cleaving SP-D. Cleavage of SP-D at physiological levels of calcium (2 mM) was similar to cleavage at 0.9 mM. Therefore, MMP-9 may play a more important role than serine proteases in regulation of SP-D function *in vivo*.

## Discussion

The data presented in this manuscript demonstrate the ability of a single protease, MMP-9, to regulate cleavage of an important innate immune regulatory molecule of the lung. Dose-dependent cleavage of SP-D by MMP-9 using levels of protein similar to that observed in patients with chronic lung disease (Figure 1) suggests *in vivo* relevance for this cleavage event. In addition, we demonstrate that this effect renders alterations in multimeric assembly (Figure 1C), and cleavage causes SP-D to no longer be able to agglutinate bacteria (Figure 2). This cleavage further affects SP-D’s innate immune functions, as bacteria are no longer efficiently phagocytosed by alveolar macrophages *in vitro* (Figure 3).

The finding that MMP-9 is able to cleave SP-D and abrogate several of its functions *in vitro* suggests an additional role of MMP-9 over-expression in disease. The interesting links that exist between these two molecules in human disease and the data presented here demonstrating their interaction suggests that the effects we saw *in vitro* may also occur in human disease. Although we only explored alterations in the innate immune functions of SP-D, these alterations could provide a potential mechanism for the increase in susceptibility to infections in COPD and CF patients. In addition, recent data has suggested the possibility of whole SP-D regulating MMP-9 release from alveolar macrophages, suggesting a potential “feed-forward” loop [48]. Whether or not MMP-9 cleaved SP-D may regulate release from alveolar macrophages is a question for further investigation.

Although previous studies have shown that several serine proteases are able to cleave SP-D, their shared cleavage site is in the CRD, and this cleavage renders SP-D unable to bind targets through its CRD [43], [44]. Contrary to this, cleavage by MMP-9 does not seem to alter CRD function (Figure 4). Therefore, the SP-D fragments produced by proteolytic cleavage may still have functions in the host, and these functions might depend on which protease produced them. Even though MMP-9 cleaved SP-D is still able to bind to carbohydrates, the overall results strongly suggest that intact, multimeric SP-D is required for optimal host-pathogen interactions.

In addition to differences in properties of the fragments produced, efficiency of cleavage is also different between serine proteases and MMP-9. While cleavage of SP-D by serine proteases is dependent on calcium concentration, cleavage by MMP-9 is unaffected by calcium. At higher concentrations of calcium, MMP-9 cleaves SP-D much more effectively than NE (Figure 5), which is the most potent serine protease for SP-D cleavage [46]. As these higher concentrations also represent physiological levels, MMP-9 may contribute as much to SP-D fragmentation *in vivo* as the serine proteases. Interestingly, the CRD of SP-D requires calcium in order to bind to carbohydrates, so in the low calcium environment at which serine proteases cleave SP-D most efficiently, the calcium might not be at optimal levels for CRD interactions with carbohydrate targets.

It is quite possible that the active cleavage of SP-D will render a novel mechanism for the pathogenesis in diseases like asthma, emphysema, and allergies, in which MMP-9 is over-expressed. A critical component of the impact of these findings in clinical disorder is to determine the extent of SP-D cleavage and dysfunction in these conditions; the use of important biomarkers reflecting this cleavage (such as determination of the amino acid cleavage fragments from lower airway secretions via mass spectrometry) may be a mechanism by which to track the loss of the intact SP-D in disease.

The potential impact of MMP-9 mediated proteolysis of SP-D may also have broad implications to the cleavage of other collectin molecules. For example SP-A, another prominent innate immune molecule, shares significant structural similarity to SP-D and may also be cleaved and inactivated by MMP-9 [30]. Identifying similarities and potential differences in the functionality of these proteins after proteolytic digestion may offer us additional insight into the roles of these collectins in human lung disorders with a protease-rich environment.

If MMP-9-mediated proteolytic loss of SP-D does indeed contribute to disease pathogenesis in clinical disease, there are potential therapeutic directions by which to target these losses of SP-D *in vivo*. One possibility is to neutralize MMP-9 with specific synthetic inhibitors or the use of endogenous antiproteases; potential questions certainly exist about tolerability and routes of delivery of these compounds [49]. If a specific cleavage site could be determined for MMP-9 cleavage of SP-D, it could be possible to mutate the cleavage site and create an MMP-9 resistant recombinant SP-D to be delivered exogenously to patients. Such a therapeutic might offer a targeted mechanism to improve bacterial clearance and regulation of lung host defense in the diseased airway.

## Materials and Methods

### Bacteria and Macrophages

MH-S cells (ATCC, Manassas, VA, USA) were maintained in RPMI 1640 (HyClone Laboratories, Logan, Utah, USA) with 10% FBS (Atlanta Biologicals, Lawrenceville, GA, USA) and 1% Penicilin-Streptomycin Solution (Mediatech, Manassas, VA, USA).

*E. coli* DH5α was grown until saturation in LB broth (Becton, Dickinson, and Company, Sparks, MD, USA) at 37°C with shaking. Stocks were made by adding glycerol and storing at −80°C.

### Cleavage of SP-D by MMP-9

Recombinant human SP-D (R&D Systems, Minneapolis, MN, USA) was diluted in DPBS with magnesium and calcium (HyClone, Logan, UT, USA) to a concentration of 20 µg/mL. Recombinant active human MMP-9 (Calbiochem, San Diego, CA, USA) was diluted in DPBS with Mg^2+^ and Ca^2+^. Cleavage reactions were carried out at 37°C. For the time course of MMP-9 digestion, reactions were stopped by adding an equal volume of 5XSDS-PAGE Sample Buffer and boiling for 5 min. For generation of fragments for use in agglutination and phagocytosis assays, the SP-D concentration was 20 µg/mL and MMP-9 was 5 µg/mL and cleavage was allowed to proceed for 4 hours and was confirmed by western blot.

### Cleavage of SP-D by NE

Cleavage of SP-D by neutrophil elastase was performed as for MMP-9. Purified NE (Enzo Life Sciences, Farmingdale, NY, USA) was incubated with 20 µg/mL of recombinant SP-D at 37°C. For generation of fragments for use in the ELISA, 10 µg/mL NE was incubated with 20 µg/mL SP-D for 1 hour at 37°C. After cleavage, a portion was analyzed by Western Blot to ensure complete cleavage of intact SP-D (data not shown).

### Western Blots

SP-D digest reactions were analyzed by SDS-PAGE under reducing conditions using 15% acrylamide gels. After transfer to a nitrocellulose membrane, blots were blocked with 5% milk, then incubated with a polyclonal antibody against SP-D diluted 1∶2,000 (R&D Systems, Minneapolis, MN, USA) for one hour at 37°C. Blots were then washed, and a 1∶30,000 dilution of anti-goat-HRP antibody was added (Abcam, Cambridge, UK) for one hour at 37°C. The blots were developed using Supersignal West Femto (Thermo, Waltham, MA, USA) and imaged using a ChemiDoc XRS (BioRad, Hercules, CA, USA).

### Native PAGE

SP-D was analyzed by native PAGE using Any KD Mini-PROTEAN TGX Gels (BioRad, Hercules, CA, USA). 0.5 µg of either intact or cleaved SP-D was mixed with RunBlue Native Sample Buffer (Expedeon, San Diego, CA) and electrophoresed in Tris-Glycine buffer. Protein was visualized using Pierce Silver Stain Kit (Thermo, Waltham, MA, USA).

### Agglutination

Frozen *E. coli* was thawed, washed, and resuspended in 1/5 original volume of DPBS with Mg^2+^ and Ca^2+^. 3.5 µL of concentrated bacteria was added to 3.5 µL of intact or cleaved SP-D at 2 µg/mL diluted in DPBS with Mg^2+^ and Ca^2+^, MMP-9 diluted in DPBS with Mg^2+^ and Ca^2+^ (not shown), or DPBS alone on a glass slide and coverslip was added. Pictures were taken 5–10 minutes after mixing.

For quantitative analysis, the OD_700_ of bacterial suspensions was monitored over time as previously described [50]. Frozen *E. coli* was thawed, washed and resuspended in PBS with intact SP-D, cleaved SP-D, or MMP-9 at an OD_700_ of ∼0.5. Measurements were taken every 10 minutes.

### Phagocytosis Assay

1×10^5^ MH-S cells per well were seeded in a 96-well plate and incubated overnight in a humid incubator at 37°C with 5% CO_2_. Bacteria were thawed, washed with HBSS (Mediatech, Manassas, VA, USA), and diluted to 1×10^7^ CFU/mL. SP-D was added to bacteria at a final concentration of 1 µg/mL and allowed to incubate at 37°C for 30 min. After washing the MH-S cells three times in assay buffer (5% FBS in HBSS), 100 µL of bacteria, bacteria with intact or cleaved SP-D, or bacteria with MMP-9 was added to wells and incubated at 37°C for 45 min. MH-S cells were washed three times and 150 µL of 2 mg/mL gentamicin sulfate was added. The plate was incubated at 37°C for 15 min, washed three times, then 100 µL of dH_2_0 was added. After 15 minutes, the lysate was mixed by thorough pipetting, and 10 µL was plated on LB agar. Agar plates were incubated overnight at 37°C and CFU counts were performed the following day.

### Fluorescence Microscopy and Flow Cytometry

*E. coli* was FITC labeled by using a modification of a previously reported protocol [51]. FITC Isomer I (Sigma, St. Louis, MO, USA) labeling solution was prepared in 0.1 M carbonate/bicarbonate buffer (pH = 9.5) at 0.03%. *E. coli* frozen stocks were thawed, washed with HBSS, then resuspended in twice their original volume of FITC labeling solution and incubated for 20 minutes at room temperature. Bacteria were washed three times, then resuspended in half their original volume of assay buffer (HBSS with 5% FBS). FITC-labeled bacteria were used in the phagocytosis assay described above, except that after washing unattached bacteria from the macrophages, cells were trypsinized, washed, and resuspended in a small amount of assay buffer (∼50 µL). For flow cytometry, MH-S cells from three wells that were given the same treatment were combined. Intact or cleaved SP-D was used in the assay at a final concentration of 1 µg/mL.

### ELISA

Purified *E. coli* K12 LPS (InvivoGen, San Diego, CA, USA) was diluted to 1 µg/mL and incubated in a 96-well E.I.A. plate at 4°C overnight (Costar, Cambridge, MA, USA). The plates were then washed 3 times using PBS with 0.1% Tween-20 (PBST), and then blocked with 5% BSA in PBS for 1 hour at 37°C. After washing 3 times, plates were incubated for 1 hour at 37°C with samples diluted 20-fold in PBST with or without 250 mM maltose. Plates were washed three times, then incubated sequentially for 2 hours at 37°C with the same primary and secondary antibodies used in the western blots described above diluted at 1∶200 and 1∶20,000, respectively. Plates were washed between antibody incubations and afterwards, then developed with TMB substrate (Sigma, St. Louis, MO, USA) for 1 hour and OD_370_ was measured on a Benchmark Plus microplate spectrophotometer (BioRad, Hercules, CA, USA).

### Statistics

All samples were run in triplicate, and results shown are representative of at least 3 independent experiments. Descriptive statistics (mean+ SD) were compared using One-Way ANOVA with Tukey’s post-hoc test. All statistical tests were performed at a 5% significance level (i.e., α = 0.05) using GraphPad Prism (La Jolla, CA).
